# Identification, Genotyping and Antimicrobial Susceptibility Testing of *Brucella* spp. Isolated from Livestock in Egypt

**DOI:** 10.3390/microorganisms7120603

**Published:** 2019-11-22

**Authors:** Aman Ullah Khan, Waleed S. Shell, Falk Melzer, Ashraf E. Sayour, Eman Shawkat Ramadan, Mandy C. Elschner, Amira A. Moawad, Uwe Roesler, Heinrich Neubauer, Hosny El-Adawy

**Affiliations:** 1 Institute of Bacterial Infections and Zoonoses, Friedrich-Loeffler-Institut, 07743 Jena, Germany; AmanUllah.Khan@fli.de (A.U.K.); falk.melzer@fli.de (F.M.); Mandy.Elschner@fli.de (M.C.E.); amira.moawad@fli.de (A.A.M.); heinrich.neubauer@fli.de (H.N.); 2 Institute for Animal Hygiene and Environmental Health, Free University of Berlin, 14163 Berlin, Germany; Uwe.Roesler@fu-berlin.de; 3 Department of Pathobiology, College of Veterinary and Animal Sciences, 35200 Jhang, Pakistan; 4 Central Laboratory for Evaluation of Veterinary Biologics, Agricultural Research Center, 11517 Abbasaia-Cairo, Egypt; tarikwaleedshell@hotmail.com; 5 Department of Brucellosis, Animal Health Research Institute, Agricultural Research Center, 12618 Dokki-Giza, Egypt; shoofa@dr.com; 6 Animal Reproduction Research Institute, Agricultural Research Center, 12556 Al Ahram-Giza, Egypt; emanramadan1311971@gmail.com; 7 Provincial Laboratory, Institute of Animal Health Research, 35516 Mansoura, Egypt; 8 Faculty of Veterinary Medicine, Kafr Elsheikh University, 33516 Kafr El-Sheikh, Egypt

**Keywords:** *Brucella*, Egypt, antimicrobial resistance, resistance-associated genes, mutation

## Abstract

Brucellosis is a highly contagious zoonosis worldwide with economic and public health impacts. The aim of the present study was to identify *Brucella* (*B*.) spp. isolated from animal populations located in different districts of Egypt and to determine their antimicrobial resistance. In total, 34-suspected *Brucella* isolates were recovered from lymph nodes, milk, and fetal abomasal contents of infected cattle, buffaloes, sheep, and goats from nine districts in Egypt. The isolates were identified by microbiological methods and matrix-assisted laser desorption ionization time-of-flight mass spectrometry (MALDI-TOF MS). Differentiation and genotyping were confirmed using multiplex PCR for *B. abortus*, *Brucella melitensis*, *Brucella ovis*, and *Brucella suis* (AMOS) and Bruce-ladder PCR. Antimicrobial susceptibility testing against clinically used antimicrobial agents (chloramphenicol, ciprofloxacin, erythromycin, gentamicin, imipenem, rifampicin, streptomycin, and tetracycline) was performed using E-Test. The antimicrobial resistance-associated genes and mutations in *Brucella* isolates were confirmed using molecular tools. In total, 29 *Brucella* isolates (eight *B. abortus* biovar 1 and 21 *B. melitensis* biovar 3) were identified and typed. The resistance of *B. melitensis* to ciprofloxacin, erythromycin, imipenem, rifampicin, and streptomycin were 76.2%, 19.0%, 76.2%, 66.7%, and 4.8%, respectively. Whereas, 25.0%, 87.5%, 25.0%, and 37.5% of *B. abortus* were resistant to ciprofloxacin, erythromycin, imipenem, and rifampicin, respectively. Mutations in the *rpo*B gene associated with rifampicin resistance were identified in all phenotypically resistant isolates. Mutations in *gyr*A and *gyr*B genes associated with ciprofloxacin resistance were identified in four phenotypically resistant isolates of *B. melitensis*. This is the first study highlighting the antimicrobial resistance in *Brucella* isolated from different animal species in Egypt. Mutations detected in genes associated with antimicrobial resistance unravel the molecular mechanisms of resistance in *Brucella* isolates from Egypt. The mutations in the *rpo*B gene in phenotypically resistant *B. abortus* isolates in this study were reported for the first time in Egypt.

## 1. Introduction

Brucellosis is considered as a common bacterial zoonotic disease of high prevalence in countries of the Middle East and the Mediterranean region, as well as some parts of Central and South America, Africa, and Asia [1,2]. Brucellosis is caused by bacteria of various species of the genus *Brucella* (*B.*) that are genetically highly related [3,4]. *Brucella* is a Gram negative, facultative intracellular pathogen classically causing infections in sheep and goats (*B. melitensis*), rams (*B. ovis*), bovines (*B. abortus*), canines (*B. canis*), pigs (*B. suis*), and rodents (*B. neotomae*) [5,6]. Brucellosis also affects terrestrial wildlife (*B. microti*) and marine mammals (*B. ceti* and *B. pinnipedialis*) [7]. However, the cross infection of animal species with brucellae has also been reported [8]. Brucellosis in livestock is causing high economic losses to livestock industry due to poor health, debility and loss of quality livestock products [9]. In humans, brucellosis causes severe acute febrile illness that becomes chronic if left untreated [10].

In developing countries, brucellosis is common but neglected disease, which has been endemic in Egypt for thousands of years and is present with a high prevalence in animals today [11,12,13,14]. Prevalence ranges from 2.47% to 26.66% in various livestock populations and this has a great socio-economic impact [15]. In Egypt, *B. abortus*, *B. suis* and *B. melitensis* strains were isolated from livestock having high levels of phylogenetic variability within each species [12]. The incidence of human brucellosis is 0.28–95 per 100,000 inhabitants per year in Egypt [16,17]. Humans get infected via the ingestion of contaminated raw milk, unpasteurized dairy products, handling of infected animals, animal discharges or dealing with *Brucella* cultures [18,19].

The diagnosis of brucellosis is still challenging and usually relies on serological tests [20], which are applied in vitro (milk or blood). Exceptionally, in vivo (allergic tests) are used. The isolation of brucellae and detection of *Brucella* DNA by PCR are the methods that allow definitive diagnosis [21].

Although confirmation of the disease is achieved by bacterial culture and identification, *Brucella* is difficult to grow and bacterial culturing is time consuming. Additionally, this method poses a risk to laboratory personnel and requires specific biosafety measures [22]. Hence, culture and biochemical typing remain the “gold standard” for the diagnosis of *Brucella* infection [23], including biochemical tests like CO_2_ requirement, H_2_S production, and dye sensitivity. Urease, oxidase, and catalase tests are also used for the typing of *Brucella* spp. [24]. A comparatively new method like matrix-assisted laser desorption ionization time-of-flight mass spectrometry (MALDI-TOF MS) has emerged for microbiological identification [25]. It is an economical, easy, rapid and accurate method based on the automated analysis of the mass distribution of bacterial proteins [26]. A recently published study indicates that MALDI-TOF MS can accurately identify 99.5% and 97% of *Brucella* strains at the genus and species level, respectively that minimizing laboratory hazards. However, there are limitations in terms of sub-species level identification [27]. *Brucella* identification and species differentiation can be accomplished using genus-specific *Brucella* PCR (B4/B5), AMOS-PCR, and Bruce-ladder PCR [28,29,30,31,32].

The intracellular location of brucellae in reticuloendothelial cells and their predilection sites (e.g., bone) limit the penetration of most antibiotics. Antimicrobial regimes with quinolones, doxycycline, rifampicin, streptomycin, and aminoglycoside alone or in combination are used to treat brucellosis [33]. Regular treatment failure and numerous reports of relapses of brucellosis following therapy exist ranging from 5% to 15% in uncomplicated cases [34]. Recently, the antimicrobial resistance in *Brucella* is emerging in brucellosis endemic regions of the world (e.g., Egypt, Qatar, Iran, Malaysia, and China) [34].

There is no proper legislation in Egypt regulating the use of antimicrobials. Some compounds such as quinolones, tetracycline, beta-lactams, aminoglycosides and imipenem are still overused non-therapeutically in Egypt to treat various human infections [35,36,37]. This improper use of antimicrobials results in the emergence of multidrug resistant bacteria [38,39,40,41]. The use of antimicrobials in farm animals to promote growth or as prophylaxis also contributes to the development of resistant bacteria and plays a key role in their spread along the food chain [42]. Antimicrobial resistance in zoonotic pathogens is an additional risk because it will limit disease treatment options in public health and veterinary settings [43]. None of the available studies highlights detailed antimicrobial susceptibility patterns of *Brucella* isolates from livestock in Egypt.

The use of antimicrobial susceptibility testing is the solution for appropriate control and treatment of brucellosis [44,45]. Micro-dilution and/or gradient strip (*E*-test) methods are used to establish minimum inhibitory concentration (MIC) for antimicrobials [45,46]. PCR assays and the subsequent sequencing of genes associated with resistance are used to identify the genetic bases of resistance [47,48,49].

Resistance to commonly used antimicrobials is mediated by mutations of *rpo*B gene (rifampicin), *gyr*A, *gyr*B, *par*C, *par*E genes (quinolones), *erm*, *mef*, *msr* (macrolides) or the presence of *tet* genes (tetracyclines), *mecA* (beta-lactams) and *flo*A (trimethoprim) [50]. Mutations in the *rpo*B and *gyr*A genes may occur naturally or can be induced in vitro [45,47,51,52].

This study aimed to isolate, identify and biotype *Brucella* strains from livestock in various regions of Egypt. Antimicrobial resistance and its genetic basis are to be investigated in the gained *Brucella* isolates.

## 2. Materials and Methods

### 2.1. Isolation and Identification

A total of 34 suspected *Brucella* isolates were recovered from clinical specimens of lymph nodes, milk and fetal stomach contents from infected cattle, buffaloes, sheep and goats located in Giza, Beheria, Asyut, Qalyubia, Beni-Suef, Ismailia, Dakahlia, and Monufia governorates/districts in Egypt (Table 1).

Bacterial isolation and identification were performed in Biological Safety Level-3 (BSL-3) laboratory. Isolates were inoculated on calf blood agar, *Brucella* medium and *Brucella* selective medium plates (Oxoid GmbH, Wesel, Germany) at 37 °C in the absence and presence of 5–10% CO2 for up to 2 weeks. Typically, round, glistening, pinpoint and honey drop-like cultures were picked and stained with Gram and modified Ziehl-Neelsen staining (MZN) methods. Subsequent biochemical tests, motility test, hemolysis on blood agar and agglutination with monospecific sera were performed [24,53]. Isolates were stored at −20 °C for further processing.

#### Identification by MALDI-TOF MS

Bacterial identification was additionally carried out using MALDI-TOF MS as described previously [27,54]. Briefly, pure cultures of suspected *Brucella* were obtained by incubating inoculated chocolate PolyViteX (PVX) agar plates (bioMérieux, Marcy-l’Étoile, France) for 48 h at 37 °C in the presence of 5% CO_2_. Samples were reliably inactivated in Biological Safety Level-3 laboratory. Approximately 10 colonies from culture medium were suspended in 50 μL of sterile HPLC water and mixed carefully. Formic acid (*v*/*v* 70%) was added for the inactivation of brucellae and for extraction of proteins. Then, 1 μL of tested sample and *Brucella* reference strains were added onto spots of a steel target plate. After inactivation, the plate was dried at room temperature followed by the addition of 0.5 μL of 100% ethanol to each well. Finally, spots were overlaid with 1 μL of reconstituted alpha-cyano-4-hydroxycinnamic acid (Bruker Daltonics, Billerica, MA, USA).

Spectra were acquired with an Ultraflex instrument (Bruker Daltonics GmbH, Bremen, Germany). Analysis was done with the Biotyper 3.1 software (Bruker Daltonics GmbH, Germany) as per the manufacturer’s instructions to exclude spectra with outlier peaks or anomalies.

Logarithmic score values (0–3.0) were determined by automatically calculating the proportion of matching peaks and peak intensities between the test spectrum and the reference spectra in the database. The identification was considered reliable when the score between 2.3 and 3.0. A logarithmic score of 1.7–2.299 was reported as ‘probable genus identification’, indicating that identification was reliable only at the genus level. When the logarithmic score was <1.7, the spectrum was reported as ‘not reliable identification’, indicating that sample could not be identified.

### 2.2. Genomic DNA Extraction and Purification

DNA was extracted from heat inactivated pure *Brucella* culture (biomass) using the HighPure PCR Template Preparation Kit (Roche Diagnostics, Mannheim, Germany) according to the manufacturer’s instructions. DNA quantity and purity were determined using a NanoDrop™ 1000 spectrophotometer (Thermo Fisher Scientific, Wilmington, USA).

### 2.3. Molecular Identification and Differentiation

The presence of the *Brucella* genus-specific *bscp31* gene [55] and *Brucella*-specific insertion sequence 711 (IS*711*) [29] was investigated for *Brucella* genus identification. Briefly, PCR was performed using 25 µL reaction mixture containing 18.3 µL HPLC water, 2.5 µL 10x PCR buffer (Genaxxon bioscience GmbH, Ulm, Germany), 1 µl of 10mM dNTP (Thermo Fisher Scientific, USA), 1 µL each forward (5′-TGG CTC GGT TGC CAA TAT CAA-3′) and reverse primer (5′ CGC GCT TGC CTT TCA GGT CTG-3′) (Jena Bioscience, Germany), 0.2 µL of 5U/µL Taq-polymerase (Genaxxon bioscience GmbH, Ulm, Germany) and 1 µL DNA template.

PCR condition was initiated by initial denaturation at 93 °C for 5 min, followed by 35 cycles of denaturation at 90 °C for 60 s, annealing at 60 °C for 60 s and elongation at 72 °C for 60 s and final elongation step at 72 °C for 5 min. PCR products (223 bp) were analyzed on 1.5% agarose gel, stained with ethidium bromide, and visualized under UV light.

The AMOS-PCR was performed to differentiate *Brucella* species [29,32] followed by a multiplex Bruce-ladder PCR assay for strain and biovar typing [30,56]. The list of primers and primer sequences for AMOS-PCR and Bruce-ladder PCR were geared from previously published [29] and [30], respectively. Briefly, for AMOS-PCR, PCR was performed using 25 µL reaction mixture containing 9.5 µL HPLC water, 12.5 µL of 2x Qiagen Master mix (Qiagen, Germany), 1 µL of 10 pmol primer mix and 2 µL DNA template. Initial denaturation at 95 °C for 5 min, was followed by 30 cycles of denaturation at 95 °C for 60 s, annealing at 58 °C for 2 min and elongation at 72 °C for 2 min and a final elongation step at 72 °C for 5 min. The Bruce-ladder PCR was performed using 12.5 µL reaction mixture containing 4.25 µL HPLC water, 6.25 µl of 2x Qiagen Master mix (Qiagen, Germany), 1 µL of 2 pmol/µL primer mix and 1 µL DNA template. Initial denaturation at 95 °C for 15 min, was followed by 25 cycles of denaturation at 94 °C for 30 s, annealing at 58 °C for 90 s, elongation at 72 °C for 3 min and a final elongation step at 72 °C for 10 min.

The PCR products from each PCR were separated by electrophoresis using 1.5% agarose gels (120 V for 60 min for conventional and AMOS-PCR and 130 V for 60 min for Bruce-ladder PCR). Gels were stained with ethidium bromide and photographed using a gene snap camera (Syngene Pvt Ltd., Cambridge, UK).

### 2.4. Antimicrobial Susceptibility Testing

The antimicrobial susceptibility of *B. melitensis* and *B. abortus* isolates was performed against eight clinically relevant antimicrobial agents (chloramphenicol, ciprofloxacin, erythromycin, gentamicin, imipenem, rifampicin, streptomycin and tetracycline) using gradient strip method (E-test, bioMerieux, Marcy L’Etoile, France) as described previously [48]. Briefly, a suspension of bacteria adjusted to 0.5 McFarland standard units was inoculated on Mueller-Hinton plates (Oxoid GmbH, Wesel, Germany) supplemented with 5% sheep blood and the gradient strips were applied. The plates were incubated at 37 °C with 5% CO_2_ for 48 h before reading. As MIC breakpoints for clinically used antimicrobials are not yet established for brucellae, the guidelines for slow-growing bacteria (*Haemophilus influenzae*) were used as an alternative [57]. Quality control assays were performed using *E. coli* (161008BR3642, DSM 1103, ATCC 25922). The susceptibility profiles of *Brucella* isolates are presented as resistant and susceptible using minimum inhibitory concentrations (MIC), MIC_50_ and MIC_90_. The interpretations were performed using CLSI (The Clinical and Laboratory Standards Institute) [57] and EUCAST (The European Committee on Antimicrobial Susceptibility Testing) [58] using the criteria for slow growing bacteria. For rifampin, the strains were also classified as intermediate (Table 2).

### 2.5. Molecular Detection of Antimicrobial Resistance-Associated Genes

The PCR assays were performed as described previously [47,49,52,59] to detect the antimicrobial resistance-associated genes, i.e., *cat*B, *gyr*A and *gyr*B, *rpo*B, *Aac* genes and *tet* genes for chloramphenicol, ciprofloxacin, rifampicin, streptomycin, gentamicin and tetracycline, respectively (Supplementary Table S1). The primers used for amplification of the *rpo*B gene were designed by using submitted sequences for the *rpo*B gene of *B. abortus* (accession number AY562181) [47]. PCR was performed using 25 µL reaction mixture containing 2x Qiagen Mastermix, 10 pmol each forward and reverse primer (Table 1) and 5 µl DNA template. PCR was carried out by initial denaturation at 95 °C for 10 min, followed by 35 cycles of denaturation at 95 °C for 45 s, annealing (temperatures for each primer are given in Table 1) for 60 s, elongation at 72 °C for 60 s and a final elongation step at 72 °C for 10 min. Twenty microliters of each reaction mixture were analyzed by gel electrophoresis (1% agarose gel with ethidium bromide).

### 2.6. PCR Amplicon Sequencing and Data Analysis

Amplified PCR products for *gyr*A, *gyr*B and *rpo*B genes were purified using Qiagen QIAquick Gel extraction kit (Qiagen, Germany) and sent for sequencing (Eurofins Genomics Germany GmbH, Ebersberg, Germany). All consensus sequences were aligned and compared to the reference *Brucella* genes obtained from NCBI for detection and evaluation of nucleotide diversity and mutations using the software Geneious^®^ R11.1.5 (https://www.geneious.com). The sequences of *gyr*A (CP034103 and AE017223), *gyr*B (CP007760 and SDWB01000001) and *rpo*B (AY562181 and AY540346) genes of *B. melitensis* and *B. abortus* were geared from Gene bank and used as reference. Amino acid sequences were determined along with nucleotide sequences to identify missense mutations using BLAST.

## 3. Results

### 3.1. Microbiological Identification

Based on microbiological and biochemical characteristics, 21 strains were typed as *B. melitensis* biovar 3, eight strains were *B. abortus* biovar 1 and five samples were identified as *Achromobacter* species (Table 1). The results of MALDI-TOF MS confirmed five isolates as *Achromobacter* species while the remaining 29 isolates were identified as *Brucella* species (Table 1).

### 3.2. Molecular Identification and Differentiation

*Brucella* DNA of 24 isolates from cattle, three from buffaloes, one from a sheep and one from a goat were amplified with the genus specific assay. AMOS-PCR and Bruce-ladder PCR differentiated these 21 isolates as *B. melitensis* (17 from cattle, two from buffaloes, 1 from a sheep and 1 from a goat) and 8 isolates as *B. abortus* (seven from cattle and one from a buffalo). All isolates were confirmed as field strains (Table 1).

### 3.3. Antimicrobial Susceptibility Profiling

The in vitro MIC values against eight antimicrobial agents of all 29 *Brucella* isolates were determined by the gradient strip method (*E*-test). The MIC values along with MIC_50_ and MIC_90_ are summarized in Table 2.

In this study, 76.19%, 19.04%, 76.19%, 66.66%, and 4.76% of the *B. melitensis* isolates were resistant to ciprofloxacin, erythromycin, imipenem, rifampicin/rifampin and streptomycin, respectively. While, 25%, 87.5%, 25%, and 37.5% of *B. abortus* isolates were phenotypically resistant to ciprofloxacin, erythromycin, imipenem and rifampicin/rifampin, respectively. All 29 *Brucella* isolates were sensitive to chloramphenicol, gentamicin, and tetracycline. Four isolates of *B. melitensis* (19.04%) and one *B. abortus* isolate showed multidrug resistance against ciprofloxacin (fluoroquinolones), erythromycin (macrolides), imipenem (carbapenems) and rifampicin (ansamycins).

### 3.4. Detection of Antimicrobial Resistance-Associated Genes and Mutations

Genes associated with antimicrobial resistance (*cat*B, *Aac* and *tet* (*tet*A, *tet*B, *tet*M and *tet*O) conferring resistance to chloramphenicol, streptomycin/gentamicin and tetracycline, respectively) were not identified either in resistant or sensitive isolates. The *gyr*A, *gyr*B and *rpo*B genes were amplified in all isolates.

Mutations in *rpo*B gene associated with a rifampicin-resistant *B. melitensis* and *B. abortus* phenotypes were detected at different positions (Table 3).

Mutations in *gyr*A gene associated with phenotypic-ciprofloxacin resistance were detected at positions 167 (ATG to AGG/methionine to arginine), 197 (CCC to CGC/proline to arginine), 202 (CGC to AGC/arginine to serine), 235 (GGT to CGT/glycine to arginine), 941 (GCC to GAC/alanine to aspartic acid), 944 (GTG to GAG/valine to glutamic acid), 944-945 (GTG to GGA/valine to glycine), 946 (GCC to TCC/alanine to serine) and 962 (AAC to ACC/asparagine to threonine) in *B. melitensis* (Table 4).

Three-point mutations were also detected in *gyr*B gene at position 1141 (AAG to GAG/Lysine to Glutamine), 1144 (ATC to CTC/Isoleucine to leucine) and 1421 (TCA to TTA/Serine to Leucine) in phenotypically resistant *B. melitensis* isolates (Table 4).

Repeated mutations were detected at positions 676, 677 (TAC to CTC/tyrosine to leucine) and 1435 (AAG to CAG/lysine to glutamine) in the *rpo*B gene of phenotypic resistant *B. melitensis* isolates while the same was recorded at position 2890 (CGT to GGT/arginine to glycine) in the *rpo*B gene of *B. abortus* isolates. No mutation was detected in *gyr*A and *gyr*B gene of *B. abortus* strains.

## 4. Discussion

Brucellosis is a zoonotic disease of public health importance and is still endemic in many countries including Egypt [17,20]. In this study, the phenotypic and molecular characterization of *Brucella* isolates from cattle, buffaloes, sheep and goats obtained from different geographical locations of Egypt was performed. Additionally, the molecular basis of antimicrobial resistance in *Brucella* isolates from Egypt is reported for the first time. These results contribute to a better understanding of geographic transmission and spread of brucellae in livestock in Egypt and pave a way for specific treatment and control of the disease in animals and as well as in humans.

For the accurate diagnosis of brucellosis, isolation of bacteria or molecular proof along with suggestive clinical signs is needed. Brucellae were isolated in this study from milk, lymph nodes and fetal stomach contents as recommended in previous reports [24,60].

Twenty-one *B. melitensis* bv3 and 8 *B. abortus* bv1 were isolated from cattle, buffaloes, sheep and goats from Giza, Beheria, Asyut, Qalyubia, Beni-Suef, Ismailia, Dakahlia and Monufia governorates. Previous reports were described previously that *Brucella* was prevailing in the country [12]. The isolation of *B. melitensis* from cattle and buffaloes in this study may be attributed to mixed farming of large and small ruminants as mentioned previously [13].

Still brucellosis is a challenge to treat in humans, particularly after delayed diagnosis of the infection. The WHO (World Health Organization) recommended treatment include high oral doses of rifampicin, doxycycline or tetracycline and trimethoprim-sulfamethoxazole. Although streptomycin and tetracycline are considered as powerful therapeutic agents against brucellosis, their higher toxicity limits their use [52,61]. Quinolones are promising alternatives to treat human brucellosis as they have good bioavailability and affinity for bone and soft tissues [51].

Only one study from Brazil reported reduced antimicrobial sensitivity in brucellae isolated from cattle [62]. However, the emergence of brucellae isolated from humans phenotypically resistant to ciprofloxacin, gentamycin, streptomycin, rifampicin and trimethoprim-sulfamethoxazole was reported in Egypt, Iran, Qatar, China, Norway and Malaysia [46,48,63,64,65]. Phenotypically rifampicin resistant *B. melitensis* isolates were also reported from Norway in imported cases from the Middle East, Asia or Africa [45]. Probable rifampicin resistance was noted in 19% of a large collection of *B. melitensis* isolates from humans in Egypt between 1999 to 2007 [65]. However, none of those isolates were investigated further to confirm the basis of resistance or reduced susceptibility.

In this study, a notable phenotypic resistance against ciprofloxacin (76.19%) was detected in *B. melitensis* strains isolated from animals. In contrast, none of the mentioned studies reported ciprofloxacin resistance in clinical isolates of humans and animals before. However, antimicrobial resistance against quinolones has been reported in in vitro studies of *B. melitensis* from Greece and France [49,52].

An alarming high number of rifampicin resistant (66.66%) *B. melitensis* isolates was found in this study. Previous reports from Egypt (19%), [65], Norway (24%) [45] and Kazakhstan (26.4%) [66] described comparatively low resistance. Hence, these findings are in agreement with previously published reports from Egypt that clearly showed an increase in antimicrobial resistance in various other human pathogens [37]. Reduced rifampicin susceptibilities in *B. melitensis* strains were also reported from Iran, Malaysia, China, and Kazakhstan [46,48,63,64,66].

The most striking finding of the present study was the emergence of phenotypic antimicrobial resistance against erythromycin (19.04%), imipenem (76.19%) and streptomycin (4.76%) in *B. melitensis* isolates. However, the increased use of these antimicrobials in Egypt in veterinary and human practices may be the cause of the emerging of this resistance [37].

The phenotypic antimicrobial resistance against ciprofloxacin (25%), erythromycin (87.5%), imipenem (25%) and rifampicin (37.5%) of *B. abortus* isolated in this study was not proved previously. Multidrug resistant strains of *B. abortus* isolated from cattle in this study were reported previously in Brazil [62]. Four isolates of *B. melitensis* and one isolate of *B. abortus* showed multidrug resistance against ciprofloxacin, erythromycin, imipenem and rifampicin. These findings are in agreement with the results of Barbosa Pauletti et al. who find corresponding resistance among *B. abortus* isolates from cattle in brazil [62]. All *B. melitensis* and *B. abortus* isolates in this study were sensitive to chloramphenicol, gentamicin and tetracycline. These findings are comparable to previously published reports in Egypt, China, Qatar and Kazakhstan [46,48,65,66].

The target for rifampicin action in *Brucella* as well as in other bacteria is the beta-subunit of the DNA dependent RNA polymerase (RNAP) encoded by *rpo*B gene [47,51]. In this study, mutations were identified in *rpo*B gene associated with phenotypic rifampicin resistant *Brucella* strains isolated from clinical specimens of animals in Egypt. Mutations were detected in all phenotypically resistant brucellae. Multiple and variable mutations were noted in each isolate along with few commonly shared mutations among many isolates. Frequent mutations at positions 676, 677-TAC to CTC (tyrosine to leucine, 38%) and 1435-AAG to CAG (lysine to glutamine, 23.8%) in the *rpo*B gene of phenotypically resistant *B. melitensis* were detected. These mutations are different from previously reported mutations (in vitro mutations) associated with rifampicin resistance in *Brucella* [47].

Johansen et al. reported mutations in phenotypic rifampicin resistant or intermediately resistant *B. melitensis* isolates [45], which in agreement with the findings of this study with additional mutations were detected as well as in intermediate rifampicin resistant *B. melitensis*.

To the best of our knowledge, this study is the first report that proved mutations in the *rpo*B gene of rifampicin resistant *B. abortus* strains. Frequent mutations were detected at position 2890-CGT to GGT (arginine to glycine, 37.5%).

Fluoroquinolone/quinolone resistance in *Brucella* is multifactorial by nature in addition to obvious mutations of the *gyr*A, *gyr*B, *par*C and *par*E genes [51,52]. In this study, the mutations in *gyr*A and *gyr*B genes in phenotypically resistant *B. melitensis* and *B. abortus* to ciprofloxacin were investigated. The mutations in *gyr*A did not correspond with fluoroquinolone resistance mutations described by Turkmani et al. [49], although they investigated mutations in vitro selected fluoroquinolone resistant *Brucella* mutants. The mutations in the *gyr*B gene detected at positions 1141-AAG to GAG (lysine to glutamine), 1144-ATC to CTC (isoleucine to leucine) and 1421-TCA to TTA (serine to leucine) of *B. melitensis* considered as novel findings of this study. None of these mutations was detected in *B. abortus* strains in *gyr*A or *gyr*B genes. However, the role of *par*C, *par*E and efflux systems cannot be ruled out for fluoroquinolone resistance [51] as we did not investigate the changes in *par*C and *par*E genes.

Genes responsible for resistance against chloramphenicol (*cat*B), gentamicin (*Aac*) and tetracycline (*tet*A, *tet*B, *tet*M and *tet*O) were not detected in all investigated *Brucella* isolated in this study, which in accordance with the phenotypic antimicrobial susceptibility results of isolated *Brucella* isolates. It is also worth mentioning that all resistant *Brucella* strains were isolated from animals and they showed resistance to antimicrobials clinically used in humans practice, suggesting that the source of these *Brucella* strains may be of human origin. These findings point to the fact that inter-species and intra-host species *Brucella* transmission is common, but spillback may occur also when chronic human brucellosis is mistreated and resistant strains are shedded [67]. A likely scenario would be the animal keeper interface.

The emergence of antimicrobial resistance (AMR) in bacteria is a public health issue globally and already compromises the treatment options regarding effectiveness of antimicrobials and control of several bacterial infections especially caused by gram-negative bacteria [68]. Wide spreading AMR in these bacteria is likely to persist and even worsen in future due to the uncontrolled use of antimicrobials. Rifampicin and ciprofloxacin are effective against intracellular bacteria like *Brucella* [33]. Higher phenotypic resistance in *Brucella* against these antimicrobials is likely to limits the treatment effectiveness, owing to the increased number of infections. Emergence of multidrug resistance *Brucella* in livestock species in this study may pose serious threat to humans as these bacteria often transferred from animals to humans through food chain [69]. Being a zoonotic pathogen and given the emergence of increased antimicrobial resistance in *Brucella* species, the situation with respect to hospital care may worsen and limits the treatment options in public health settings.

## 5. Conclusions

Brucellosis is a contagious and often communicable worldwide zoonosis with high morbidity and low mortality. There has been a tremendous increase in inter host-species infection in the recent decades, especially in developing countries when farm animal species are kept on the same premises without biosecurity precautions. The disease is endemic in Egypt and *B. melitensis* and *B. abortus* have been reported as the main causative agents of brucellosis in humans and animals. High phenotypic resistance against ciprofloxacin, erythromycin, and imipenem were detected in *Brucella* spp. isolated from different districts and animals species reflecting a broad geographical distribution. The molecular identification of mutations in antimicrobial resistance associated genes highlight the mechanism of resistance in *Brucella* spp. There is a need for further insights into the epidemiology and spread of antimicrobial resistant *Brucella* in Egypt. The WHO regimes have to be reevaluated and awareness among physicians about AMR needs to be raised.

## Figures and Tables

**Table 1:** Microbiological and molecular identification of *Brucella* spp. isolated from animal species in Egypt.

Sample ID	Animal Species	Origin of Sample	Type of Sample	Growth with CO_2_			Slide Agglutination A-M-R-Serum				MALDI-TOF MS	Molecular Identification
				^c^Bruc	^d^Brusel	^e^BBA	A	M	R	Result		
18RB17227	Cattle	Giza	Lymph node	+	+	+	^a^+ve	+ve	^b^−ve	*B. melitensis 3*	*Brucella* spp. (*B. abortus*)	*B. melitensis*
18RB17228	Cattle	Giza	Lymph node	+	+	+	+ve	+ve	−ve	*B. melitensis 3*	*Brucella* spp. (*B. abortus*)	*B. melitensis*
18RB17229	Cattle	Giza	Lymph node	+	+	+	+ve	+ve	−ve	*B. melitensis 3*	*Brucella melitensis*	*B. melitensis*
18RB17230	Cattle	Giza	Lymph node	+	+	+	+ve	+ve	−ve	*B. melitensis 3*	*Brucella* spp. (*B. melitensis*)	*B. melitensis*
18RB17231	Cattle	Giza	Lymph node	+	+	+	−ve	−ve	−ve	* NA	*Achromobacter* spp.	-ve
18RB17232	Cattle	Giza	Lymph node	+	+	+	−ve	−ve	−ve	NA	*Achromobacter* spp.	-ve
18RB17233	Cattle	Giza	Lymph node	+/−	+/−	+/−	+ve	−ve	−ve	*B. abortus 1*	*B. abortus*	*B. abortus*
18RB17234	Cattle	Giza	Lymph node	+	+	+	−ve	−ve	−ve	NA	*Achromobacter* spp.	-ve
18RB17235	Cattle	Giza	Lymph node	+	+	+	+ve	+ve	−ve	*B. melitensis 3*	*Brucella* spp. (*B. microti*)	*B. melitensis*
18RB17236	Cattle	Giza	Lymph node	+	+	+	+ve	+ve	−ve	*B. melitensis 3*	*Brucella* spp. (*B. melitensis*)	*B. melitensis*
18RB17237	Cattle	Giza	Lymph node	+	+	+	−ve	−ve	−ve	NA	*Achromobacter* spp.	-ve
18RB17238	Cattle	Giza	Lymph node	+	+	+	+ve	−ve	−ve	*B. abortus 1*	*Brucella* spp. (*B. microti*)	*B. melitensis*
18RB17239	Cattle	Giza	Lymph node	+	+	+	−ve	−ve	−ve	NA	*Achromobacter* spp.	-ve
18RB17240	Cattle	Beheira	Lymph node	+	+	+	+ve	+ve	−ve	*B. melitensis 3*	*Brucella* spp. (*B. microti*)	*B. melitensis*
18RB17241	Cattle	Beheira	Lymph node	+	+	+	+ve	+ve	−ve	*B. melitensis 3*	*Brucella* spp. (*B. microti*)	*B. melitensis*
18RB17242	Cattle	Beheira	Lymph node	+/−	+/−	+/−	+ve	−ve	−ve	*B. abortus 1*	*B. abortus*	*B. abortus*
18RB17243	Cattle	Beheira	Lymph node	+/−	+/−	+/−	+ve	−ve	−ve	*B. abortus 1*	*B. abortus*	*B. abortus*
18RB17244	Buffalo	Asyut	Lymph node	+	+	+	+ve	+ve	−ve	*B. melitensis 3*	*Brucella* spp. (*B. abortus*)	*B. melitensis*
18RB17245	Buffalo	Asyut	Lymph node	+/−	+/−	+/−	+ve	−ve	−ve	*B. abortus 1*	*B. abortus*	*B. abortus*
18RB17246	Goat	Beni-Suef	Lymph node	+	+	+	+ve	+ve	−ve	*B. melitensis 3*	*Brucella* spp. (*B. microti*)	*B. melitensis*
18RB17247	Cattle	Asyut	Lymph node	+	+	+	+ve	+ve	−ve	*B. melitensis 3*	*Brucella* spp. (*B. melitensis*)	*B. melitensis*
18RB17248	Cattle	Qalyubia	Lymph node	+	+	+	+ve	+ve	−ve	*B. melitensis 3*	*Brucella* spp. (*B. microti*)	*B. melitensis*
18RB17249	Cattle	Qalyubia	Lymph node	+	+	+	+ve	+ve	−ve	*B. melitensis 3*	*Brucella* spp. (*B. melitensis*)	*B. melitensis*
18RB17250	Sheep	Beni-Suef	Lymph node	+	+	+	+ve	+ve	−ve	*B. melitensis 3*	*Brucella* spp. (*B. melitensis*)	*B. melitensis*
18RB17251	Cattle	Beni-Suef	Lymph node	+	+	+	+ve	+ve	−ve	*B. melitensis 3*	*Brucella* spp. (*B. microti*)	*B. melitensis*
18RB17252	Cattle	Ismailia	Lymph node	+	+	+	+ve	+ve	−ve	*B. melitensis 3*	*Brucella* spp. (*B. melitensis*)	*B. melitensis*
18RB17253	Cattle	Ismailia	Lymph node	+	+	+	+ve	+ve	−ve	*B. melitensis 3*	*Brucella* spp. (*B. abortus*)	*B. melitensis*
18RB17254	Cattle	Ismailia	Lymph node	+	+	+	+ve	+ve	−ve	*B. melitensis 3*	*Brucella* spp.	*B. melitensis*
18RB17255	Cattle	Beheira	Fetal stomach content	+/−	+/−	+/−	+ve	−ve	−ve	*B. abortus 1*	*B. abortus*	*B. abortus*
18RB17256	Cattle	Dakahlia	Lymph node	+/−	+/−	+/−	+ve	−ve	−ve	*B. abortus 1*	*B. abortus*	*B. abortus*
18RB17257	Cattle	Monufia	Lymph node	+/−	+/−	+/−	+ve	−ve	−ve	*B. abortus 1*	*B. abortus*	*B. abortus*
18RB17258	Cattle	Monufia	Milk	+	+	+	+ve	+ve	−ve	*B. melitensis 3*	*Brucella* spp. (*B. abortus*)	*B. melitensis*
18RB17259	Cattle	Qalyubia	Lymph node	+/−	+/−	+/−	+ve	−ve	−ve	*B. abortus 1*	*B. abortus*	*B. abortus*
18RB17260	Buffalo	Qalyubia	Lymph node	+	+	+	+ve	+ve	−ve	*B. melitensis 3*	*Brucella* spp. (*B. microti*)	*B. melitensis*

* NA-not applicable, ^a^ Positive, ^b^ Negative, ^c^ *Brucella* medium, ^d^ *Brucella* selective medium, ^e^ *Brucella* blood agar.

**Table 2:** Antimicrobial resistance profiles of 21 *B. melitensis* and 8 *B. abortus* isolated from livestock species in Egypt against 8 clinically used antibiotics using *E*-test. Breakpoint and Minimal Inhibitory Concentration (MIC_50_, MIC_90_) for *B. melitensis* and *B. abortus* used in this study according to CLSI and EUCAST recorded for *H. influenzae* [57,58] were provided.

Antibiotic	Class	Breakpoints			*B. melitensis*			*B. abortus*		
		Sensitive (mg/L)	Intermedium (mg/L)	Resistant (mg/L)	R (%)	MIC_50_ (mg/L)	MIC_90_ (mg/L)	R (%)	MIC_50_ (mg/L)	MIC_90_ (mg/L)
Chloramphenicol	Phenicols	≤2	4	≥8	0.0	1	2	0.0	0.25	0.5
Ciprofloxacin	Fluoroquinolones	≤0.06	−	>0.06	76.19	0.12	0.25	25.0	0.06	0.06
Erythromycin	Macrolides	−	−	≥16	19.04	4	8	87.5	32	32
Gentamicin	Aminoglycosides	−	−	≤4	0.0	11	11	0.0	0.12	0.5
Imipenem	Carbapenems	≤2	−	>2	76.19	8	8	25.0	1	4
Rifampicin	Ansamycins	≤1	2	≥4	66.66	4	8	37.5	2	4
Streptomycin	Aminoglycosides	−	−	≤16	4.76	1	2	0.0	0.25	0.5
Tetracycline	Tetracyclines	≤2	4	≥8	0.0	0.06	0.12	0.0	0.03	0.12

−. Not determined

**Table 3:** Detection of mutations in *rpo*B gene associated with rifampicin resistance in *B. melitensis* and *B. abortus*.

ID	*Brucella* spp.	RIF Resistance	Mutation Sites	Mutation	Amino Acid Change	NCBI (Accession No.)
18RB17227	*B. melitensis*	4	676, 677 1816 1818 1820, 1822 1824, 1825 1826, 1828 1829, 1831 1835, 1837 1838 1842, 1843	TAC to CTC GAT to GAA GTC to GCC GTT to ATA TAC to TTT CTG to GTT TCG to GAC ATG to GGC GAA to AAA GAA to GGT	Tyrosine to leucine Aspartic acid to glutamic acid Valine to alanine Valine to isoleucine Tyrosine to phenylalanine Leucine to valine Serine to aspartic acid Methionine to glycine Glutamic acid to lysine Glutamic acid to glycine	MN544028, MN544042, MN544056, MN544070, MN544084
18RB17228	*B. melitensis*	4	676, 677 3901, 3902	TAC to CTC TAC to ACC	Tyrosine to leucine Tyrosine to threonine	MN544029, MN544043, MN544057, MN544071, MN544085
18RB17229	*B. melitensis*	4	676, 677 1011 1456, 1458 1787 2491	TAC to CTC AAC to AGC GAA to AAG AAG to ACG ACC to CCC	Tyrosine to leucine Asparagine to serine Glutamic acid to lysine Lysine to threonine Threonine to proline	MN544030, MN544044, MN544058, MN544072, MN544086
18RB17230	*B. melitensis*	8	676, 677 1435 1798, 1799 1801, 1802 1804, 1806 1807 2209, 2210	TAC to CTC AAG to CAG GGC to AAC AAG to GGG GTG to CTT ACG to TCG ATC to TCC	Tyrosine to leucine Lysine to glutamine Glycine to asparagine Lysine to glycine Valine to leucine Threonine to serine Isoleucine to serine	MN544031, MN544045, MN544059, MN544073, MN544087
18RB17235	*B. melitensis*	>8	676, 677 1469	TAC to CTC GTC to GGC	Tyrosine to leucine Valine to glycine	MN544032, MN544046, MN544060, MN544074, MN544087
18RB17236	*B. melitensis*	8	676, 677	TAC to CTC	Tyrosine to leucine	MN544033, MN544047, MN544061, MN544075, MN544089
18RB17238	*B. melitensis*	16	677 1780 1786, 1788 2869, 2871	TAC to TTC TAT to GAT AAG to CAA CGT to GGG	Tyrosine to phenylalanine Tyrosine to aspartic acid Lysine to glutamine Arginine to glycine	MN544034, MN544048, MN544062, MN544076, MN544090
18RB17240	*B. melitensis*	16	2494, 2496	TCG to CTC	Serine to leucine	MN544035, MN544049, MN544063, MN544077, MN544091
18RB17241	*B. melitensis*	6(8)	1435 2870, 2871	AAG to CAG CGT to CCG	Lysine to glutamine Arginine to proline	MN544036, MN544050, MN544064, MN544078, MN544092
18RB17246	*B. melitensis*	4	676, 678 1436, 1437 2870 3898 3901	TAC to CTT AAG to ACA CGT to CCT TAC to AAC ACG to CCG	Tyrosine to leucine Lysine to threonine Arginine to proline Tyrosine to asparagine Threonine to proline	MN544037, MN544051, MN544065, MN544079, MN544093
18RB17249	*B. melitensis*	4	1435, 1437 2170 2203, 2205 2869 3152, 3153 3154, 3156 3157	AAG to GTA GGC to CGC ATC to TTT CGT to GGT GTG to GGT CAG to GCA CGC to AGC	Lysine to valine Glycine to arginine Isoleucine to phenylalanine Arginine to glycine Valine to glycine Glutamine to alanine Arginine to serine	MN544038, MN544052, MN544066, MN544080, MN544094
18RB17253	*B. melitensis*	4	1435 1745	AAG to CAG GCC to GGC	Lysine to glutamine Alanine to glycine	MN544039, MN544053, MN544067, MN544081, MN544095
18RB17258	*B. melitensis*	6	676, 677 2501, 2502	TAC to CTC CAC to CCA	Tyrosine to leucine Histidine to proline	MN544040, MN544054, MN544068, MN544082, MN544096
18RB17260	*B. melitensis*	4	1435 3670, 3672	AAG to CAG CAG to TAT	Lysine to glutamine Glutamine to tyrosine	MN544041, MN544055, MN544069, MN544083, MN544097
18RB17233	*B. abortus*	4	703, 704 709, 710 1457, 1458 1460 2512 2515, 2517 2890, 2892 3123 3124, 3125	ACT to CTT ACC to CAC AAG to ACA GAA to GGA ACC to CCC TCG to CTC CGT to GGG GAC to GAG GAC to ATC	Threonine to leucine Threonine to histidine Lysine to threonine Glutamic acid to glycine Threonine to proline Serine to leucine Arginine to glycine Aspartic acid to glutamic acid Aspartic acid to isoleucine	MN544013, MN544016, MN544019, MN544022, MN544025
18RB17242	*B. abortus*	>4	698, 699 1457, 1458 1460 1789 1801 2887 2890	TAC to TTT AAG to ACA GAA to GGA ATC to GTC TAT to GAT GAG to AAG CGT to GGT	Tyrosine to phenylalanine Tyrosine to threonine Glutamic acid to glycine Isoleucine to valine Tyrosine to aspartic acid Glutamic acid to lysine Arginine to glycine	MN544014, MN544017, MN544020, MN544023, MN544026
18RB17245	*B. abortus*	4	709 2890	ACC to CCC CGT to GGT	Threonine to proline Arginine to glycine	MN544015, MN544018, MN544021, MN544024, MN544027

**Table 4:** Detection of mutations in *gyr*A and *gyr*B genes associated with ciprofloxacin resistance in *B. melitensis.*

ID	*Brucella* spp.	CIPResistance	Gene	Mutation Sites	Mutation	Amino Acid Change	NCBI (Accession No.)
18RB17230	*B. melitensis*	0.5	*gyr*A	167 197 202 235	ATG to AGG CCC to CGC CGC to AGC GGT to CGT	Methionine to arginine Proline to arginine Arginine to serine Glycine to arginine	MN536677
18RB17235	*B. melitensis*	0.25		944, 945 946	GTG to GGA GCC to TCC	Valine to glycine Alanine to serine	MN536678
18RB17238	*B. melitensis*	0.25		941 944	GCC to GAC GTG to GAG	Alanine to aspartic acid Valine to glutamic acid	MN536679
18RB17254	*B. melitensis*	0.12		962	AAC to ACC	Asparagine to threonine	MN536680
18RB17230	*B. melitensis*	0.5	*gyr*B	1144	ATC to CTC	Isoleucine to leucine	MN536681
18RB17244	*B. melitensis*	0.25		1141	AAG to GAG	Lysine to Glutamine	MN536682
18RB17252	*B. melitensis*	0.12		1421	TCA to TTA	Serine to Leucine	MN536683
18RB17254	*B. melitensis*	0.12		1421	TCA to TTA	Serine to Leucine	MN536684

## Supplementary Materials

The following are available online at https://www.mdpi.com/2076-2607/7/12/603/s1, Table S1: List of primers and primer sequences used for detection of antimicrobial associated resistance mechanism.
