# Supplementary material for: Identification, Genotyping and Antimicrobial Susceptibility Testing of Brucella spp. Isolated from Livestock in Egypt
Source: Microorganisms. 2019 Nov 22;7(12):603. doi: 10.3390/microorganisms7120603 (PMC6955977; doi:10.3390/microorganisms7120603)
Supplement: Supplementary file 1 [file microorganisms-07-00603-s001.pdf]

Table S1. List of primers and primer sequences used for detection of antimicrobial associated resistance mechanism.

Antibiotic	Method	Primer	Primer Sequence (5'–3')	Annealing Temp. (°C)	Amplicon length (bp)	Reference
Rifampin	(*rpo*B) PCR	*rpo*B_M1-2	Fr- CAA GAC TGT CAC CTA TAC CCG T<br>Rw- TGC GGA TAT ACG CAC CGA TAT	63	400	This study
		*rpo*B_M3	Ft- AGT ATC GCG TCG GTC TGC TCC GC<br>Rw- ATC GAC AAC CTT GCG ATA CG	60	401	This study
		*rpo*B_M4	Fr- GCA TGG AAC CGA TCG TCG<br>Rw- GAA TCG AGG TGA ACA CGT CG	63	400	This study
		*rpo*B_M5	Fr- GAA ATC GAG CGT CTG GCC AA<br>Rw- TGG ATC TTG CGC TTC ACA G	58	400	This study
		*rpo*B_M6	Fr- CCG GTC TTC GAC GGT GCG G<br>Rw- CTG CAG CGT GTA GGC CGC	60	300	This study
		+354rB<br>-720rB	Fr- TGC GAA GTC CAT CAA GGA CAT<br>Rw- ACG GGT ATA GGT GAC AGT CTT G	60	367	[49]
Quinolones	*gyr*A PCR	+4651Bru_*gyr*A<br>-5589Bru_*gyr*A	Fr- TGC AGC GGT CTT ATC TTG ATT<br>Rw- CAA ACG AGG TCT GCA AAG G	55	1000	[53]
	*gyr*B PCR	*gyr*B QRDR	Fr- GTT GTC GAG AAG GTC ATT CAG G<br>Rw- GCG TTG AAG CCG TGC GTT TC	55	360	[54]
Tetracyclines	*tet*(O) PCR	*tet*O	Fr- TTC TGG GCT TCT GTC GGG TTG T<br>Rw- CTA TTC GGG CGG CGG GGT T	60	557	[61]
	tet(M) PCR	*tet*M	Fr- CGA GGT CCG TCT GAA CTT TGC G<br>Rw- GCG GCA CTT CGA TGT GAA TGG T	60	583	
	tet(A) PCR	*tet*A	Fr- TCG TCG CCG CCC TGA TGG<br>Rw- GCC GCA TAG ATC GCC GTG AAG	60	451	
	tet(B) PCR	*tet*B	Fr- ATC GGG TCC CTG GTA GCA ATG G<br>Rw- AGT CCT CCG CAA AGG GTT CCA A	60	371	
Chloramphenicol	*cat*(B) PCR	*cat*B	Fr- AGC CTC TCA GCG AAC AGG TCA A<br>Rw- AGT CCC ACC ACG CCA TTT CCA	60	507	[61]
Aminoglycosides	*Aac* PCR	*Aac*	Fr- TGG AGC ACT GGC GCG GAT T<br>Rw- GAA AAC CAC GGG CGA ACT GTC A	60	200	[61]
	Aac(6')-Ib PCR	*Aac(6')-Ib*	Fr- AGA AGA AGC ACG CCC GAC ACT<br>Rw- GTT CCC AAG CCT TTG CCC AGT T	60	237	
	Aac(3)-Ia PCR	*Aac(3)-Ia*	Fr- GAG TTC GGA GAC GTA GCC ACC T<br>Rw- ATA GAG AGC CAC TGC GGG ATC G	60	324	
	Aac(6)-Ib-cr4 PCR	*Aac(6)-Ib-cr4*	Fr- CAA CAG CAC CGA TTC CGT CAC A<br>Rw- GTT CCC AAG CCT TTG CCC AGT T	60	339	
	Ant(3)-Ia PCR	*Ant(3)-Ia*	Fr- TCA GAG GTA GTT GGC GTC ATC G<br>Rw- GCG GCG AGT TCC ATA GCG TTA	60	490	
